# A physiological comparison of three techniques for reviving sockeye salmon exposed to a severe capture stressor during upriver migration

**DOI:** 10.1093/conphys/cov015

**Published:** 2015-04-21

**Authors:** Graham D. Raby, Samantha M. Wilson, David A. Patterson, Scott G. Hinch, Timothy D. Clark, Anthony P. Farrell, Steven J. Cooke

**Affiliations:** 1 Fish Ecology and Conservation Physiology Laboratory, Department of Biology and Institute of Environmental Science, Carleton University, Ottawa, Canada K1S 5B6; 2 Fisheries and Oceans Canada, Science Branch, Pacific Region, Cooperative Resource Management Institute, School of Resource and Environmental Management, Simon Fraser University, Burnaby, Canada V5A 1S6; 3 Pacific Salmon Ecology and Conservation Laboratory, Department of Forest and Conservation Sciences, University of British Columbia, Vancouver, Canada V6T 1Z4; 4 Australian Institute of Marine Science, PMB 3, Townsville, QLD 4810, Australia; 5 Department of Zoology and Faculty of Land and Food Systems, University of British Columbia, Vancouver, Canada V6T 1Z4

**Keywords:** Bycatch, catch and release, discards, exhaustive exercise, post-release mortality, stress response

## Abstract

Capture of fish in commercial and recreational fisheries causes disruption to their physiological homeostasis and can result in delayed mortality for fish that are released. For fish that are severely impaired, it may be desirable to attempt revival prior to release to reduce the likelihood of post-release mortality. In this study, male sockeye salmon (*Oncorhynchus nerka*) undergoing their upriver migration were used to examine short-term physiological changes during the following three revival treatments after beach seine capture and air exposure: a pump-powered recovery box that provided ram ventilation at one of two water flow rates; and a cylindrical, in-river recovery bag, which ensured that fish were oriented into the river flow. Beach seine capture followed by a 3 min air exposure resulted in severe impairment of reflexes such that fish could not maintain positive orientation or properly ventilate. All three revival treatments resulted in significant reductions in reflex impairment within 15 min, with full recovery of reflex responses observed within 60–120 min. For most variables measured, including plasma lactate, cortisol and osmolality, there were no significant differences among revival treatments. There was some evidence for impaired recovery in the low-flow recovery box, in the form of higher haematocrit and plasma sodium. These data mirror published recovery profiles for a recovery box study in the marine environment where a survival benefit occurred, suggesting that the methods tested here are viable options for reviving salmon caught in freshwater. Importantly, with most of the benefit to animal vitality accrued in the first 15 min, prolonging recovery when fish become vigorous may not provide added benefit because the confinement itself is likely to serve as a stressor.

## Abstract (precis)

We used physiological measurements to compare different methods for reviving salmon after a severe capture stressor that involved 3 min of air exposure. Both a powered recovery box and a flow-through in-river recovery bag enabled revival, but prolonged confinement appeared to act as an additional stressor.

## Introduction

The fate of fish released from recreational and commercial fisheries is a concern in many systems (Davis, 2002; Cooke and Schramm, 2007) because post-release mortality is cryptic and can impede conservation and management efforts (Coggins *et al*., 2007; Baker and Schindler, 2009; Gilman *et al*., 2013). Correspondingly, researchers have made efforts to quantify the effects of different capture and handling processes (Davis, 2002) and to evaluate mitigation options (Uhlmann and Broadhurst, 2013), with physiological measures commonly being used in such studies as objective measures of animal welfare (Diggles *et al*., 2011; Cooke *et al*., 2013; Donaldson *et al*., 2013; Wilson *et al*., 2014). Fisheries capture is an acute stressor that causes a neuroendocrine stress response, anaerobic exercise and often some degree of asphyxiation (e.g. through air exposure) or exposure to hypoxic water during crowding. These stressors combine to result in a disruption to homeostasis that has been well characterized (e.g. Wells *et al*., 1986; Davis *et al*., 2001; Marcalo *et al*., 2006). Depending on the intensity of the stressor, physiological disturbance can be significant enough to cause immediate mortality (Chopin *et al*., 1996), or fish that fail to regain homeostasis can suffer delayed mortality hours or days after release (Parker *et al*., 1959; Wood *et al*., 1983; Davis, 2002; Skomal, 2007).

**Figure 1:**
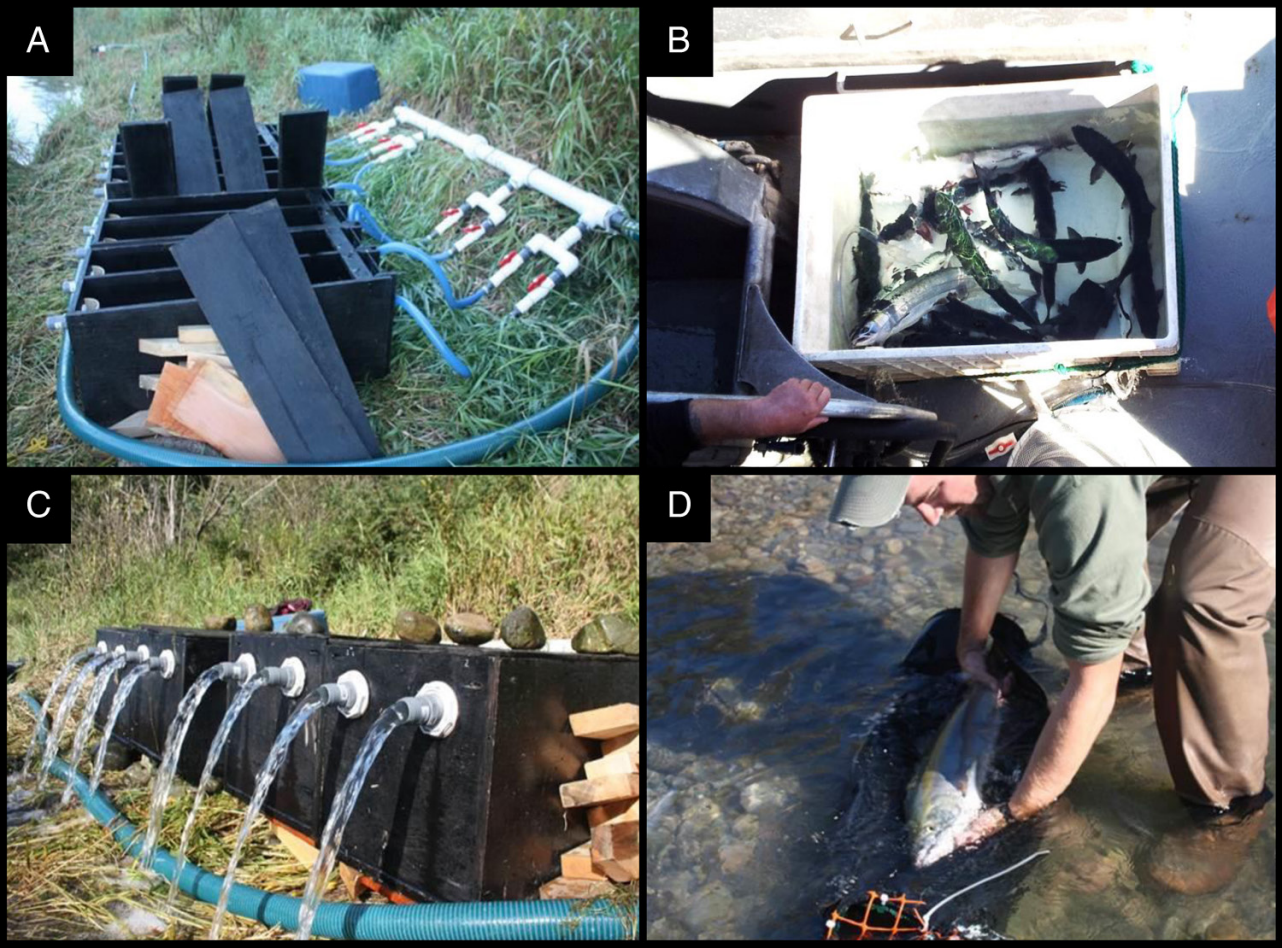
Photographs of the Fraser boxes set up at the study site with lids off, not in operation (**A**) and the outlet (downstream) end of the Fraser boxes during operation, containing fish, during the high-flow treatment (0.9 l s^−1^; **C**). Also shown is a photograph of a standard ‘recovery tote’ used to revive fish in commercial marine seine fisheries (**B**; not tested in this study but see Raby *et al*., 2015) and a photograph of the fish recovery bag at the surface prior to submergence for the revival treatment (**D**; additional photograph in Raby *et al.*, 2014a). Photographers: Jude Isabella (A, C and D) and Paul Brajcich (B).

Efforts to revive visibly lethargic fish prior to release are sometimes used in fisheries as means of reducing the likelihood of post-release mortality. For example, some recreational anglers will manually move fish back and forth or in a figure-of-eight pattern to promote flow across the gills (ram ventilation); these techniques are recommended by some management agencies despite a lack of experimental support (Pelletier *et al*., 2007; Robinson *et al*., 2013). Some commercial fisheries employ ‘recovery totes’ (Fig. 1B) that provide a safe on-board recovery environment for bycatch before it is discarded (Farrell *et al*., 2001a). In catch-and-release recreational fisheries, flow-through recovery bags (Fig. 1D) can be used to reduce impairment of fish before release (Brownscombe *et al*., 2013), which may be particularly useful where there is a threat of post-release predation (Cooke and Philipp, 2004; Raby *et al*., 2014b).

Facilitated revival techniques may be relevant in upriver-migrating Pacific salmon (*Oncorhynchus* spp.), where fisheries usually target a single species that co-migrates with others, resulting in capture and release of non-target salmon species and some portion of the target species (Gale *et al*., 2011; Raby *et al*., 2014a). If a capture stressor is severe (e.g. via long angling times, air exposure, crowding or asphyxiation in nets), the resulting level of impairment can be high enough that fish exhibit negative orientation (drift downstream) and irregular ventilation patterns (Raby *et al*., 2012; Brownscombe *et al*., 2013) and can show a delay in upstream migration (Makinen *et al*., 2000; G. D. Raby, unpublished data). In the Fraser River (British Columbia, Canada), post-release migration failure has been shown to reach 20–40% (Donaldson *et al*., 2013; Raby *et al*., 2014a) despite a fisheries policy objective to release non-target fish in an ‘unharmed’ state (DFO, 2001). In the marine environment, a revival device known as the Fraser box was validated and can now be used as a way of promoting physiological recovery and short-term survival in coho salmon (*Oncorhynchus kisutch*) bycatch (Farrell *et al*., 2001a). The Fraser box offers the advantage of a strong water current directed at the head of the fish (ram ventilation), which allows for effective revival of fish that have ceased to ventilate by themselves, something not offered by the ‘recovery totes’ that have been used in commercial salmon fisheries (Farrell *et al*., 2001a).

In freshwater, comparisons of the Fraser box, a comparatively portable recovery bag and traditional manual techniques (i.e. holding fish facing into the flow upon release) suggest that the benefits of facilitated revival techniques are highly context dependent (Donaldson *et al*., 2013; Robinson *et al*., 2013; Nguyen *et al*., 2014; Raby *et al*., 2014a). Active swimming in free-flowing water has been reported to promote rapid recovery and suppress the cortisol response in exhaustively exercised hatchery rainbow trout (*Oncorhynchus mykiss*; Milligan *et al*., 2000) and in troll-caught coho salmon (Farrell *et al*., 2001b), though it provided no benefit to the recovery of angled largemouth bass (*Micropterus salmoides*; Suski *et al*., 2007). However, if a salmon is unable to swim, maintain orientation or ventilate after a severe capture stressor, immediate release to the wild would risk short-term mortality via respiratory failure (Farrell *et al*., 2001a; Skomal, 2007) and make them vulnerable to predators (Quinn and Buck, 2001; Forrest *et al*., 2009; Raby *et al*., 2014b). Thus, facilitated revival may reduce mortality only when the severity of the capture stressor and resulting level of impairment are high (Farrell *et al*., 2001a; Donaldson *et al*., 2013; Raby *et al*., 2014a).

The purpose of the present study was to compare short-term physiological recovery profiles among three revival treatments following seine capture of sockeye salmon (*Oncorhynchus nerka*) and 3 min of air exposure. The study was motivated by a need to assess different options for reviving salmon from severe capture stress during upriver migration. Although beach seine capture was used, we expect the general trends to be transferable to other fisheries in which strenuous exercise (e.g. for 3–5 min) is followed by extended air exposure (e.g. 2–3 min), such as in recreational fisheries. Indeed, a previous study found no difference in the physiological status of sockeye salmon between fish caught by angling and beach seining (without air exposure; Donaldson *et al*., 2011). We exclusively used male salmon to avoid data being confounded by sex because circulating cortisol is higher and more variable in females (Baker and Vynne, 2014). In addition to assessing physiological status via blood measures, we used an objective and quantitative assessment of whole-animal condition and vitality [reflex action mortality predictors (RAMP); Davis, 2010] both before and during the recovery. One revival treatment was a lightweight, inexpensive and portable in-river flow-through fish bag. The two other revival treatments used a specially designed recovery box (which requires a powered pump to generate flow) but with two different water flow rates (i.e. 0.2 and 0.9 l s^−1^).

## Materials and methods

All experimental procedures were approved by the University of British Columbia Animal Care Committee (Animal Care #A08-0388) and Carleton University Animal Care Committee (B09-10) in accordance with guidelines set by the Canadian Council on Animal Care.

### Study site and capture treatment

Capture of sockeye salmon occurred from 20 to 23 September 2010 on the Harrison River (49°17′5.8″ N, 121°54′27.4″ W), a large tributary of the Fraser River (British Columbia, Canada). Water temperature was ∼14–15°C for the duration of the experiment. The salmon had already migrated 115 river km upstream from ocean entry and were probably a mix of Harrison River and Weaver Creek populations (population origin was not identified), both of which spawn within 5 km of each other. Peak spawning activity occurs in mid-October for Weaver Creek sockeye salmon and mid-November for the Harrison River population; therefore, fish were about mid-way through their development of secondary sexual characteristics, and sex was externally identifiable. Fish were caught using a beach seine (90 m long × 9 m deep × 5 cm diamond stretch mesh) that was pulled to shore but left in sufficient water depth (∼60 cm) that crowding was minimized to allow fish to swim around in the enclosed net. Fewer than 50 salmon were caught in each net haul, but only up to four fish were used from each net so that capture and handling times could be kept consistent (i.e. the four fish were all removed within 1 min of the net being pulled into shore). The capture process elicited some burst swimming and lasted 3–4 min from when the net was deployed to when it was pulled into the beach. Male sockeye salmon were visually identified and dip netted for transfer into wetted black fish bags (cylindrical, 20 cm diameter, 1 m length) made of Hypalon (thick synthetic rubber). The fish bags had lengthwise zippers that allowed fish to be enclosed, as well as 4 cm diamond mesh on each end to allow water to flow through the bags when they were submerged (Fig. 1D). The bags were first pulled onto the riverbank to expose fish to air for 3 min, a duration chosen to ensure maximal reflex impairment, i.e. fish would drift downstream upside down if released without a revival treatment. During both dip netting and aerial confinement in the fish bags, the salmon exhibited further burst-type exercise. Thus, the capture stressor involved a combination of air exposure and exercise. At the completion of air exposure, the fish bags containing salmon were submerged in the river for the initial RAMP assessment (see next subsection) and prior to transfer to revival treatments.

### Reflex assessments, revival treatments and physiological sampling

Reflex action mortality predictors have been validated as a vitality indicator for several species, including sockeye salmon (Donaldson *et al*., 2012; Raby *et al*., 2012, 2013; Brownscombe *et al*., 2013; Cooke *et al*., 2014). To perform the initial RAMP assessment (Fig. 1D), the individual fish bags were gently pulled to the surface, opened and the fish removed. The RAMP assessments checked for the presence of five reflexes known to be present in vigorous individuals, and they required ≤15 s to complete. The five reflexes were as follows: tail grab (whether the fish responded to the handler grabbing its tail with a burst forward); body flex (whether the fish vigorously attempted to struggle free of a handler holding it out of water around the centre of its body); vestibular-ocular response (whether the fish's eye rolled to track the handler when it was rolled on its side out of the water); head complex (whether the fish exhibited a regular pattern of ventilation when held just above the water surface); and orientation (whether the fish could right itself within 3 s when turned upside down in the water column). Individual reflexes were recorded as impaired if the handler was unsure whether they were present or absent. The RAMP scores calculated for each individual represent the proportion of reflexes that were impaired (absent).

After the initial RAMP assessment, fish were assigned to one of three revival treatments. The first treatment involved placing individual fish back into the fish bags and submerging the bags 10 m from the riverbank, where water depth was 1 m. The bags were affixed to a metal rod that had been driven into the riverbed, and the flow of water at the site of bag attachment was ∼10 cm s^−1^. Attenuation of water velocity within the bag was ∼10% (measurement reported by Donaldson *et al*., 2013), meaning that the water velocity inside the bag was ∼9 cm s^−1^, which converts to ∼2.8 l s^−1^ based on the diameter of the bag (20 cm).

The other two revival treatments involved the use of Fraser boxes. Four Fraser boxes were used (built to match Farrell *et al*., 2001a) and each had two fish compartments (each 20 cm wide × 40 cm deep × 90 cm long) with a 2.54 cm diameter inflow valve that could be adjusted to provide the two treatment water flows of 0.2 and 0.9 l s^−1^ (outflow of the boxes from the higher flow rate is shown in Fig. 1C). The outflow valve (Fig. 1C) at the opposite end was the same in all the boxes. The Fraser boxes were set up along the riverbank and continuously supplied with fresh river water by a gasoline-powered pump. Fish were placed in the boxes facing into the flow, and the lids were replaced and secured to prevent fish being able to jump out if they were revived.

Separate groups of fish remained in the revival treatments for 15, 30, 60 or 120 min (*n* = 7–11 fish per sampling time and revival treatment; average = 9) before being reassessed for RAMP, sacrificed by cerebral percussion and sampled for blood by caudal puncture using a 21-gauge needle and a ­heparinized vacutainer (3 ml with lithium heparin; BD, Franklin Lakes, NJ, USA). Whenever revival resulted in fish that were too ­vigorous to allow reflex assessment, fish were simply assigned an unimpaired status (RAMP score = 0; following Raby *et al*., 2012).

An additional 10 salmon were assessed and sampled as above within 1 min of landing of the seine without air exposure or RAMP assessment (which results in further brief handling and air exposure), and nine more salmon were sampled immediately after the 3 min air exposure (i.e. time zero).

### Blood analyses

Blood samples were placed immediately into an ice-water slurry and analysed within 15 min for haematocrit (Hct; as a percentage using haematocrit tubes centrifuged at 10 000***g*** for 5 min) and haemoglobin (Hb; in grams per litre using a hand-held meter calibrated for fish blood; HemoCue Hb 201^+^; HemoCue, Ängelholm, Sweden; Clark *et al*., 2008). Mean corpuscular haemoglobin content (MCHC) was calculated for each fish as [Hb]/(Hct/100). Whole blood was then centrifuged for 5 min at 7000***g*** so that plasma could be separated and stored in liquid nitrogen before later transfer to a −80°C freezer.

In the laboratory, blood plasma was thawed and assessed for sodium and potassium (Cole-Palmer, model 410 single-channel flame photometer), chloride (Haake Buchler digital chloridometer), cortisol (Neogen enzyme-linked immunosorbent assay with Molecular Devices Spectramax 240pc plate reader), osmolality (Advanced Instruments 3320 freezing-point osmometer), lactate and glucose (YSI 2300 Stat Plus analyser) using methods previously described (Farrell *et al*., 2001a). The metrics chosen allowed us to assess the response and recovery of osmoregulatory status (osmolality, chloride, potassium and sodium), indicators of stress (cortisol, glucose and lactate) and oxygen transport capacity of the blood (haematocrit, haemoglobin and MCHC).

### Data analysis and statistics

The main objective of our analysis was to assess whether recovery profiles differed among the three revival treatments. Tests were conducted on blood variables using two-factor analysis of variance (ANOVA using type III sums of squares), with revival treatment and revival time as fixed effects and with interactions removed if non-significant. Tukey's HSD *post hoc* comparisons followed ANOVAs where needed (family-wise α of 0.05). In most cases, there was no effect of treatment group (i.e. revival technique; see *Results*), so a second ANOVA was conducted with treatment removed as a factor and two time points added: at-capture pre-air exposure and time zero (i.e. immediately after air exposure). These one-way ANOVAs were carried out to assess general physiological changes over time and whether recovery was taking place (i.e. return towards control levels) across treatment groups. To guard against type I errors, α was set at a conservative 0.0023 (11 response variables including RAMP scores, two main tests for each, one including time-zero pre-revival data and one without, as described above). The Shapiro–Wilk test and Levene's test were used to assess assumptions of normality and heteroscedasticity, respectively, along with manual examination of the plotted data. Plasma sodium and RAMP scores did not meet parametric assumptions (after attempts at transformations in the case of the former), so Kruskal–Wallis ANOVAs were used to test effects of time and treatment separately, with Kruskal–Wallis *post hoc* multiple comparisons tests used where appropriate (family-wise α of 0.05). All statistical tests were conducted using RStudio (version 0.98.953; RStudio, Inc., Boston, MA, USA; http://www.rstudio.com/). Data are presented as means ± SEM. Data associated with this paper are publicly archived in figshare (http://dx.doi.org/10.6084/m9.figshare.1333432).

## Results

**Figure 2:**
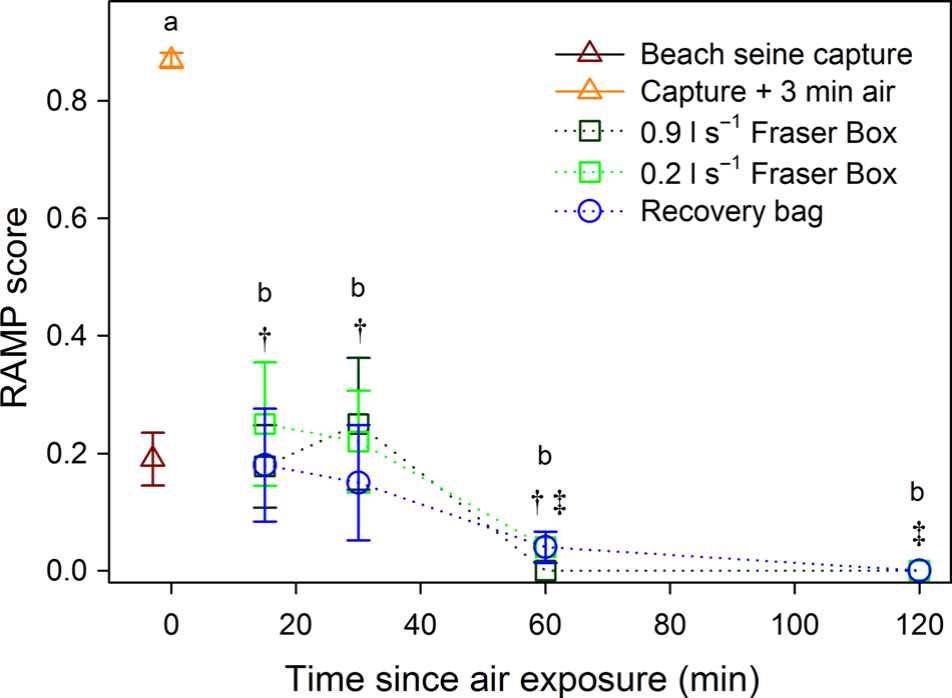
Mean reflex action mortality predictors (RAMP) scores (±SEM) for sockeye salmon upon beach seine capture (data from Donaldson *et al*., 2012), after the addition of 3 min of air exposure and after different durations of recovery in three revival treatments. All data points represent separate groups of fish (i.e. no repeat sampling), and higher scores represent more impaired fish (see *Methods* for full details on the reflex assessment). There were no significant differences among revival treatments (revival time points pooled, Kruskal–Wallis ANOVA, χ^2^ = 1.44, d.f. = 2, *P* = 0.49), but grouping fish by time point (including time zero, i.e. immediately post-air exposure) revealed a significant effect of time (χ^2^ = 178.2, d.f. = 4, *P* < 0.001), with dissimilar letters indicating significant *post hoc* differences (Kruskal–Wallis *post hoc* multiple comparisons). Focusing on a comparison among revival durations, there was a significant effect of time (χ^2^ = 27.0, d.f. = 3, *P* < 0.001) and significant *post hoc* differences that are shown by dissimilar symbols. Standard error bars are not present at 120 min because all fish had RAMP scores of 0 (likewise for the 0.9 l s^−1^ treatment at 60 min).

After beach seine capture, all fish were responsive, exhibited positive orientation and were ventilating regularly (i.e. RAMP scores of ∼0.2; see Fig. 2). An addition of 3 min of air exposure resulted in most fish becoming unresponsive; 46% exhibited complete loss of reflexes (RAMP score = 1.0) and a further 45% lost four of five reflexes (0.8). The former group would be characterized as either moribund or dead in a normal commercial fishery setting (Farrell *et al*., 2001a), while the one reflex retained in the latter group was the vestibulo-ocular response.

All combinations of revival treatments and durations significantly decreased RAMP scores compared with those in fish sampled immediately following 3 min of air exposure (dissimilar letters in Fig. 2; Kruskal–Wallis ANOVA, effect of time, χ^2^ = 178.1, d.f. = 4, *P* < 0.001; effect of revival treatment, χ^2^ = 1.4, d.f. = 2, *P* = 0.48), with no significant *post hoc* differences among revival durations. Thus, independent of the treatment type, most of the benefit to fish vitality accrued in the first 15 min of recovery (Fig. 2). Indeed, of the 28 fish revived for 15 min, 71% could be described as vigorous (RAMP score = 0–0.2) and 93% were self-ventilating. Excluding time zero fish (post-air exposure) from the analysis to focus on the different revival durations, there was a significant overall effect of revival duration (χ^2^ = 27.1, d.f. = 3, *P* < 0.001), with impairment significantly lower after 120 min (at which time no reflex impairment was observed) than in the 15 and 30 min groups (*post hoc* differences shown by dissimilar symbols in Fig. 2).

Of the 121 immobile and poorly ventilating fish that were air exposed, 97.5% were revived by the recovery treatments. The three fish that died exhibited negative orientation at the outset of revival treatment (two in the 120 min group of the 0.9 l s^−1^ Fraser box; one in the 30 min group of the 0.9 l s^−1^ Fraser box) and faced into the front corners of the chamber rather than directly into the inflow valve. Other fish were regularly observed making vigorous attempts to burst free of the Fraser boxes prior to their predetermined sampling times.

**Figure 3:**
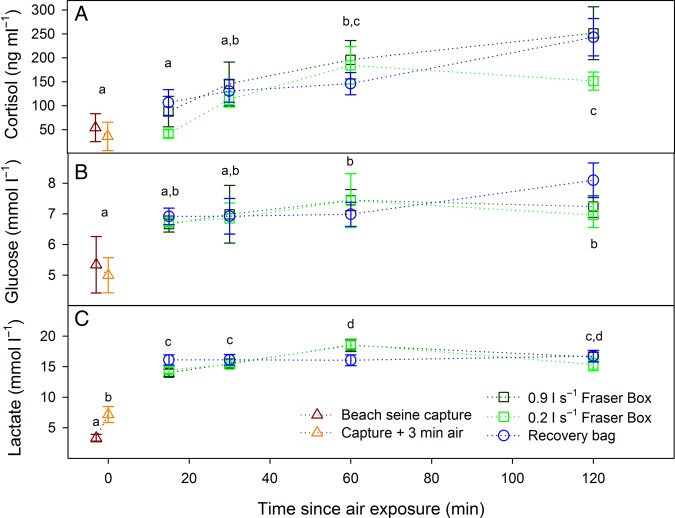
Mean ± SEM plasma cortisol (**A**), glucose (**B**) and lactate (**C**) in sockeye salmon before and after 3 min of air exposure and after different durations in three revival treatments that followed. Significant overall main effects of time occurred for cortisol, glucose and lactate (see *Results*), and dissimilar letters indicate significant *post hoc* differences among time points using Tukey's HSD.

**Figure 4:**
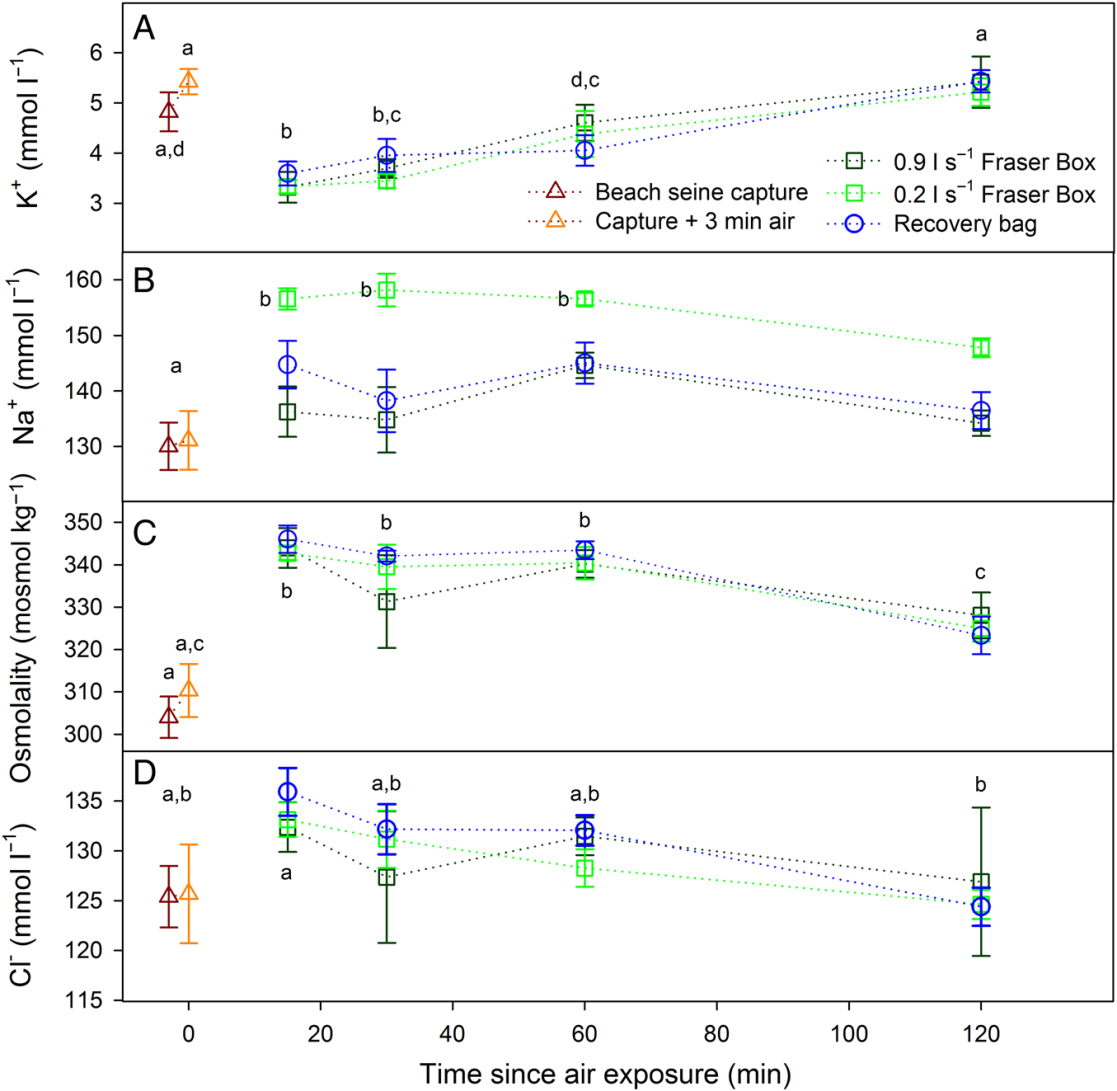
Mean ± SEM plasma potassium (**A**), sodium (**B**), osmolality (**C**) and chloride (**D**) for fish captured by beach seine, then air exposed for 3 min, followed by different durations in one of three revival treatments. Significant overall effects of time occurred for time for each variable (see *Results*; shown by dissimilar letters), and for sodium (B) a significant effect of treatment, with the 0.2 l s^−1^ treatment having significantly higher values than the other two treatments when comparing only within those four time points (see *Results* for statistics). Dissimilar letters in B show the data points that diverged significantly from control (pre- and post-air exposure) values, based on pairwise *post hoc* comparisons among all data points shown in B (Kruskal–Wallis *post hoc* multiple comparisons tests).

**Figure 5:**
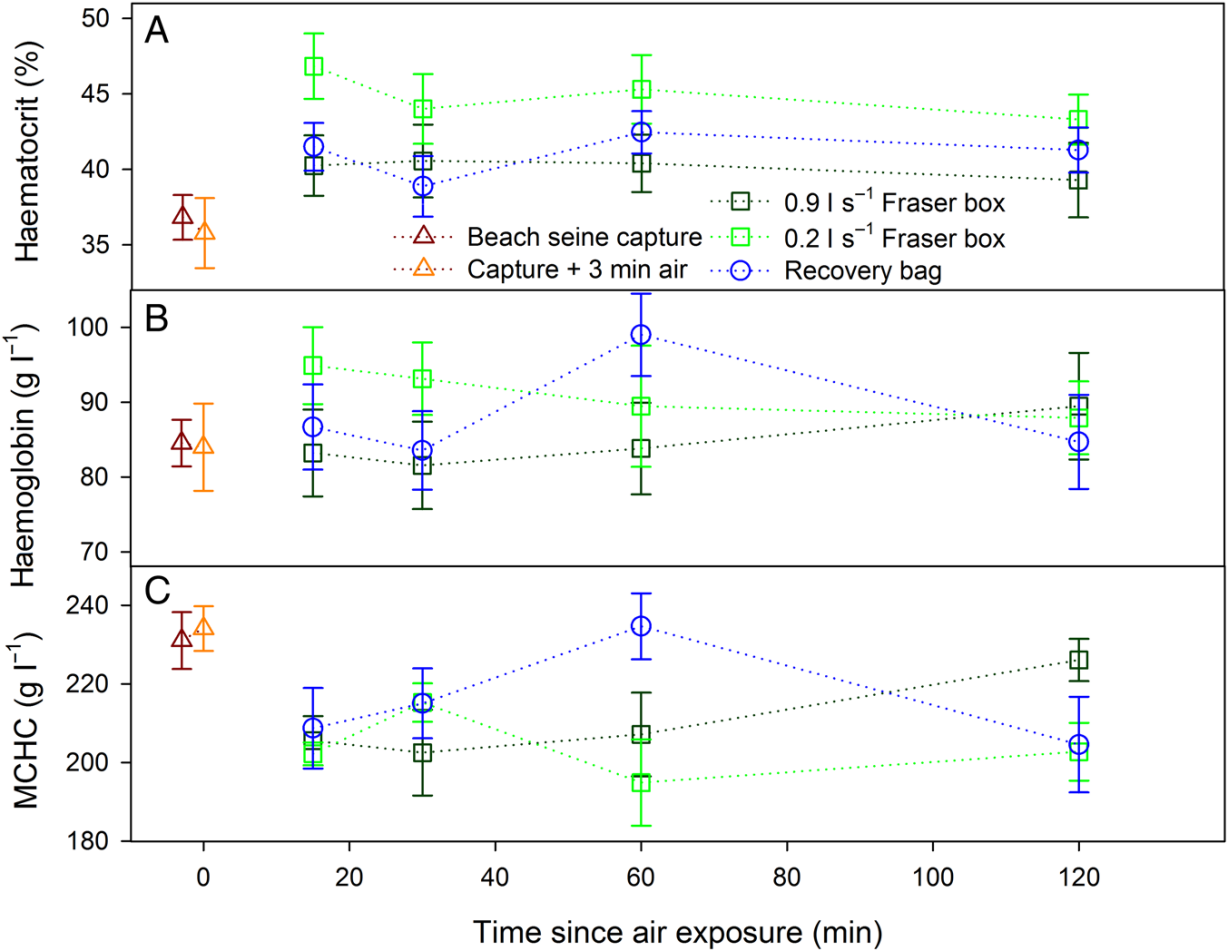
Mean ± SEM haematocrit (Hct), haemoglobin (Hb) and mean corpuscular haemoglobin content (MCHC) measured from whole blood in the field in fish sampled upon capture, with the addition of 3 min of air exposure and followed by different durations of recovery in three revival treatments. There was a significant overall effect of revival treatment on haematocrit (but not of time; *post hoc* differences explained in *Results*). The MCHC was calculated from [Hb]/(Hct/100).

Most blood variables exhibited significant changes across time points, but there were few differences among revival treatments. Air exposure significantly increased lactate, which then further increased during revival and remained elevated across revival durations, exhibiting few further changes (*post hoc* differences shown in Fig. 3C; overall effect of time, *F*_5,123_ = 67.02, *P* < 0.001). The main effect of treatment was not significant for lactate (*F*_2,102_ = 0.08, *P* = 0.91). Plasma cortisol and glucose exhibited similar patterns, with significant overall effects of time (cortisol, *F*_5,121_ = 8.65, *P* < 0.001; and glucose, *F*_5,123_ = 4.78, *P* < 0.001) but not treatment (both *P* > 0.10), and a tendency for an increase throughout revival, especially for cortisol (*post hoc* differences among time points shown in Fig. 3A and B). During revival, plasma potassium decreased sharply from time zero to 15 min (Fig. 4A; overall effect of time, *F*_5,123_ = 16.86, *P* < 0.001) and then increased towards time-zero levels with increasing durations of revival, but the type of revival treatment used had no effect (*F*_2,102_ = 0.33, *P* = 0.72). Plasma chloride and osmolality exhibited opposite patterns to that of potassium, with an increase from control levels by 15 min, followed by a decrease with increasing time (overall effect of time for chloride, *F*_5,123_ = 2.76, *P* = 0.02; and for osmolality, *F*_5,123_ = 19.78, *P* < 0.001), and no differences among treatments (*P* > 0.60 for both variables; Fig. 4). There were no statistically significant effects on haemoglobin or MCHC with respect to time or treatment (all *P* > 0.03; Fig. 5).

Although there were no differences among revival treatments for most variables, a notable difference among groups occurred for plasma sodium (Fig. 4B), with higher concentrations across the four revival durations in the low-flow (0.2 l s^−1^) Fraser box treatment (overall effect of treatment, χ^2^ = 38.4, d.f. = 2, *P* < 0.001). *Post hoc* comparisons with revival durations pooled confirmed that plasma sodium was significantly higher in the 0.2 l s^−1^ Fraser box treatment than in the other two treatments during recovery (*P* < 0.05 in both cases). Kruskal–Wallis ANOVA multiple comparisons with fish grouped by time and treatment (i.e. all groups shown in Fig. 4B) indicated that the 0.2 l s^−1^ Fraser box group was the only treatment to increase plasma sodium significantly above control levels (dissimilar letters in Fig. 4B; overall Kruskal–Wallis ANOVA, χ^2^ = 64.94, d.f. = 13, *P* < 0.001). Fish in the 0.2 l s^−1^ Fraser box treatment also had the highest haematocrit, although that effect did not reach significance (Fig. 5A; effect of time, *F*_3,103_ = 0.73, *P* = 0.67; and effect of treatment, *F*_2,103_ = 5.63, *P* = 0.003). However, with time removed as a factor and controls added as a fourth treatment, there were significant differences (*F*_3,125_ = 9.32, *P* < 0.001), and *post hoc* comparisons revealed that: (i) mean haematocrit for the 0.2 l s^−1^ Fraser box was higher than the control group and 0.9 l s^−1^ fish; (ii) haematocrit for recovery bag fish was significantly higher than control fish but not different from the other two revival treatments; and (iii) 0.9 l s^−1^ Fraser box fish were not different from control fish.

## Discussion

This study confirms that the Fraser box and recovery bag can both be used to achieve short-term revival of Pacific salmon after a capture stressor with extended air exposure incurred during upriver migration. Our experiment lacked true controls in the form of physiological data from fish sampled after being released to the river, and such data would be virtually impossible to obtain (see Cooke *et al*., 2013 for candid discussion of challenges with physiological sampling), but there are existing data against which ours can be compared (see below). Moreover, we caution that although immediate revival was achieved (97.5% of cases), we did not evaluate whether the revival treatments affected post-release survival or spawning success. Nevertheless, all three treatments showed evidence of being effective methods for reviving fish that, after the capture stressor we imposed, were so impaired that many of them would have been characterized as dead, asphyxiated or moribund in a fishery setting (i.e. RAMP scores of 0.8–1.0; Farrell *et al*., 2001a). The RAMP scores recorded after 3 min of air exposure (0.8–1.0) were at or above the upper limit of those recorded in endangered coho salmon caught in Fraser River beach seine fisheries, at which subsequent post-release mortality was very high (>70%) relative to fish with low RAMP scores (∼20%; Raby *et al*., 2014a). In such cases, there may be the potential to increase post-release survival using revival techniques, but that possibility remains to be evaluated. The vestibular-ocular response is always the final reflex to become impaired with increasing stressor intensity (Raby *et al*., 2013); salmon without this reflex (46% of those in this study) ostensibly exhibit no signs of life. Thus, in some instances there may be opportunities to revive and release fish that otherwise would be retained because of a presumption that mortality has already occurred.

An important discovery was that after merely 15 min in a revival treatment, 71% of fish were vigorous and ventilating and were capable of some burst exercise (i.e. tail grab or body flex reflexes). In contrast, coho salmon caught in purse seines and revived in industry-standard recovery totes (Fig. 1B) showed no significant improvement in RAMP scores irrespective of revival duration (Raby *et al*., 2015), indicating that not all revival methods are effective. While the present study did not assess post-release survival, the same revival methods have demonstrably improved survival in other contexts. The original validation of the Fraser box in the marine ­environment noted a reduction in 24 h mortality from 57 to 6.5% for ‘asphyxiated’ gillnet-caught fish (i.e. RAMP scores of 1.0) in a comparison with a traditional recovery tote (‘blue box’; Farrell *et al*., 2001a). Indeed, the short-term mortality rate observed in the present study (2.5% overall) was similar to that seen by Farrell *et al*. (2001a). For sockeye salmon that were angled and air exposed for 1 min in the lower Fraser River, 15 min of recovery bag revival almost doubled post-release survival (from 28.6 to 50.0%; Donaldson *et al*., 2013). Conversely, substantial data have now accumulated that suggest these revival treatments do not benefit post-release survival of salmon after mild or moderate capture stressors, i.e. fish that are able to maintain orientation following the capture stressor (i.e. RAMP scores <0.6; Donaldson *et al*., 2013; Robinson *et al*., 2013; Nguyen *et al*., 2014; Raby *et al*., 2014a). Indeed, manually holding salmon facing into flow for 1 min after moderate capture stressors may even reduce post-release survival in laboratory experiments (Robinson *et al*., 2013) and field ­studies (Robinson *et al.*, 2015). Those findings are important because they imply that the utility of revival gear is context dependent. We concur with the earlier suggestion for commercially caught coho salmon that a vigorous fish should be released immediately to prevent additional stress (Farrell *et al*., 2001a), such as that observed here, with fish struggling to escape during prolonged recovery periods. Thus, revival treatments that are most likely to promote physiological recovery and minimize additional stress may be those in which fish are revived via ram ventilation until the moment they regain positive orientation and regular ventilation and thereafter released to continue their recovery in the river (Donaldson *et al*., 2013; Raby *et al*., 2014a). Such a treatment could be possible with recovery gears modified with a viewing window so that the handler is able to assess the condition of the animal visually without repeatedly subjecting it to physical assessment (i.e. to determine RAMP scores). However, in contexts where the risk of post-release predation is high, the locomotory benefit of a more extended revival treatment (e.g. 60 vs. 15–30 min; Fig. 2) would perhaps outweigh the drawback of added confinement stress (Brownscombe *et al*., 2013; Cooke *et al*., 2014).

The present recovery data for physiological variables can be compared with those of Farrell *et al*. (2001a), where salmon were in similarly poor condition prior to Fraser box revival in saltwater. The blood variables here exhibited similar patterns, with immediate increases in osmolality, sodium, chloride, lactate, glucose and cortisol (Figs 3 and 4; Table 1 of Farrell *et al*., 2001a). There were important quantitative ­differences for the variables between the two studies. For example, plasma lactate was higher in the previous study (Table 1 of Farrell *et al*., 2001a) after 1 h in Fraser boxes for gillnet-caught fish (24.2 vs. 17.7 mmol l^−1^), as were osmolality (379.5 vs. 341.3 mosmol kg^−1^) and haematocrit (50.1 vs. 42.7%), while plasma glucose was similar (7.7 and 7.3 mmol l^−1^). These differences may reflect a variety of factors, including species differences (coho vs. sockeye salmon), sex differences (mixed vs. males only), salinity differences, maturation states (silver-bodied vs. approaching spawning), the time elapsed between initiation of the capture stressor and when blood was drawn, or the nature of the capture stressor (Cooke *et al*., 2013; Baker and Vynne, 2014; Donaldson *et al*., 2014).

Patterns in haematocrit, and especially in plasma sodium, suggested a possible limitation of the lower flow Fraser box treatment. Haematocrit increases from resting values of 20–25% (Milligan and Wood, 1986; Sandblom *et al*., 2009) almost instantaneously upon initiation of a stressor because of a massive release of catecholamines (adrenaline and noradrenaline), which trigger a splenic contraction that increases the number of circulating erythrocytes (Wendelaar Bonga, 1997). Indeed, it was clear that upon landing the beach seine the haematocrit had already been elevated (to ∼37% of blood volume). Further elevations in haematocrit appear to have occurred primarily through erythrocyte swelling rather than by an increase in number, given that MCHC tended to decrease and haemoglobin exhibited no clear patterns with respect to time or treatment (Fig. 5). During hypoxia or exhaustive exercise, an increase of arterial CO_2_ partial pressure beyond a threshold causes the release of catecholamines, which bind to β-adrenoreceptors on erythrocyte membranes (Reid *et al*., 1991), activating an Na^+^–H^+^ antiporter that shifts protons to the extracellular fluid (plasma) in exchange for sodium ions (Fievet *et al*., 1988; Perry and Gilmour, 2006). That ion exchange allows erythrocytes to maintain an internal pH favourable to the oxygen-binding affinity of haemoglobin, but causes erythrocyte swelling because water enters the cell along with sodium (Borgese *et al*., 1987; Motais *et al*., 1992). If efflux of CO_2_ and influx of O_2_ at the gills was impaired in fish in the low-flow treatment because of a lack of strong ram ventilation after lamellae collapse, they may have experienced added or prolonged elevation of arterial CO_2_ partial pressure. Carbon dioxide is mainly carried in the blood as HCO_3_^−^ with a dissociated proton (H^+^), causing increased pH, referred to as a respiratory acidosis (Wood, 1991). If a larger respiratory acidosis or catecholamine response was present in fish in the low-flow treatment, those differences may have elicited greater erythrocyte swelling and higher haematocrit.

As in the previous study on coho salmon (Farrell *et al*., 2001a), it is unclear whether the persistent physiological changes during recovery were a result of a natural time course or a result of recurrent stress as revived fish struggled to escape from the recovery treatment. Plasma lactate, glucose and cortisol continued to increase or remained elevated across revival durations in all three treatments; evidence that, at first glance, would suggest physiological recovery was not taking place. Nevertheless, those trends are in line with previous studies that show that these variables peak 0.5–2 h post-stressor (Milligan, 1996; Clark *et al*., 2012), which includes during revival treatments that ultimately benefit survival in maturing Pacific salmon (Farrell *et al*., 2001a; Donaldson *et al*., 2013) and in male sockeye salmon exposed to a chase and air exposure stressor (Donaldson *et al*., 2014). Nevertheless, there is good evidence that wild fish, particularly migrating adult salmon, are stressed by short-term confinement (Farrell *et al*., 2001a; Portz *et al*., 2006; Donaldson *et al*., 2011; Raby *et al*., 2015), and we expect that was the case in the present study. Moreover, a confinement-induced elevation in cortisol appears to slow the clearance of plasma lactate after exercise and capture stressors (Milligan *et al*., 2000; Farrell *et al*., 2001b), suggesting that the sustained elevation of plasma lactate in the present study may have been partly an artefact of confinement.

Differences in recovery profiles for the three revival treatments were absent for most variables we measured. The differences that did occur suggest that the low-flow Fraser box treatment resulted in somewhat greater physiological disturbance or impeded physiological recovery. The most consistent difference was the elevated plasma Na^+^ for the low-flow Fraser box treatment, but it is unclear what caused this because there are few comparable results elsewhere in the literature. For fish in freshwater, plasma ions normally increase after hypoxia or exhaustive exercise as a result of haemoconcentration when water content is drawn away from the blood by intracellular acidosis in muscle cells (Milligan, 1996; Kieffer, 2000). However, the few studies that have compared physiological recovery among different recovery environments have observed similar patterns in Na^+^ and Cl^−^ post-stressor (e.g. Farrell *et al*., 2001b; Donaldson *et al*., 2013), whereas in the present study the Na^+^:Cl^−^ ratio was significantly higher in the low-flow Fraser box treatment. Sodium is important for regulation of acid–base balance because it is readily exchanged for protons via membrane exchangers that operate on red blood cells and muscle cells and at the gills (Heisler, 1989; Perry and Gilmour, 2006). To satisfy electroneutrality with an elevated Na^+^:Cl^−^ ratio, some other (unmeasured) anion(s) must have increased in concentration, perhaps partly HCO_3_^−^. A larger or more sustained respiratory acidosis in blood plasma for the low-flow treatment may have triggered a larger increase in plasma sodium, perhaps via transfer of Na^+^ ions from a white muscle compartment that experienced relatively less intracellular acidosis than in other studies where fish were swum fully to exhaustion. Fish in the present study did exercise during netting but remained vigorous until removed from the net for air exposure, indicating that they had not exercised maximally (Fig. 2). This suggests that the subsequent acidosis in plasma was likely to be more respiratory than metabolic in origin relative to previous exhaustive exercise studies. Indeed, plasma lactate, which is extruded from exhausted white muscle and is the primary source of metabolic protons (Wood, 1991), did not reach maximal levels in the present study (20–25 mmol l^−1^; Farrell *et al*., 2001a). Interestingly, van Raaij *et al*. (1996) found that rainbow trout exposed to severe hypoxia that subsequently died exhibited a significantly higher Na^+^:Cl^−^ ratio in plasma during the post-hypoxia period in the lead up to death. As in the present study, the authors were unable to determine the source of the elevated Na^+^:Cl^−^ ratio given the complex nature of ion regulation, but noted that dramatic changes in ionic concentrations are maladaptive from a stress-coping standpoint (van Raaij *et al*., 1996; Wendelaar Bonga, 1997). Perturbations in plasma ions have elsewhere been linked to mortality in salmonids (Wood *et al*., 1983; Jeffries *et al*., 2011), and large changes in ion concentrations can affect the structure and function of macromolecules and directly damage tissues via osmotic swelling (Moyes and Schulte, 2008).

In summary, from the perspective of small-scale freshwater fisheries, a key finding here is that lightweight and inexpensive recovery bags immersed in the flow of a river can generate the same physiological recovery profiles as the previously validated Fraser box (Farrell *et al*., 2001a), which is a heavy, expensive and non-portable device better suited to commercial fishing boats. Much is known about ways to minimize capture stress (e.g. Davis, 2002; Cooke and Suski, 2005), but in cases where angling times are long, if fish are air exposed for detangling or photography or become asphyxiated in small-scale gillnet fisheries, recovery bags may represent a useful tool for fishers interested in reviving fish prior to release, particularly for fish faced with conservation problems (e.g. interior Fraser coho salmon; Raby *et al*., 2014a). Our presumption is that the recovery bags require flow to provide a revival benefit, and thus would mainly be of use in fisheries that occur in riverine environments. Future experiments should clarify thresholds at which revival treatments can benefit physiological recovery and survival. The capacity of fish to recover from acute stressors has clear relevance to fitness, and as such, represents a fascinating intersection of comparative physiology and ecology (Ricklefs and Wikelski, 2002; Donaldson *et al*., 2010). Further experiments comparing the effects of different recovery environments on physiological responses can help to generate knowledge that is of fundamental interest and equally relevant to fisheries management and conservation.

## Funding

The research was funded by a Natural Sciences and Engineering Research Council (NSERC) Strategic grant (to S.J.C., S.G.H. and A.P.F.). S.J.C. and A.P.F. were also supported by the Canada Research Chairs program. G.D.R. was supported by an NSERC scholarship and an Ontario Graduate Scholarship.
